# The influence of HLA matching on graft survival in lung transplant recipients is indication specific: a UNOS database analysis

**DOI:** 10.1183/13993003.01890-2025

**Published:** 2026-09-08

**Authors:** Afolarin A. Otunla, Kumaran Shanmugarajah, Ghazel Mukhtar, Sarah Nabulsi, Daniel Dolan, Justin Salciccioli, Dominic C. Marshall, Maria Lucia Madariaga, Joseph Shalhoub

**Affiliations:** 1 Division of Surgery and Interventional Science, University College London, London, UK; 2 Department of Surgery, University of Chicago, Chicago, IL, USA; 3 Imperial College School of Medicine, Imperial College London, London, UK; 4 Department of Life Sciences, Imperial College London, London, UK; 5 Department of Critical Care, Brigham and Women's Hospital, Boston, MA, USA; 6 Department of Surgery and Cancer, Imperial College London, London, UK; 7 Academic Section of Vascular Surgery, Department of Surgery and Cancer, Imperial College London, London, UK; 8 Imperial Vascular Unit, Imperial College Healthcare NHS Trust, London, UK

## Abstract

**Background:**

Lung transplantation offers definitive treatment for end-stage pulmonary disease, but graft survival remains inferior to other solid organ transplants. A key driver of graft loss is antibody-mediated rejection triggered by donor–recipient human leukocyte antigen (HLA) mismatch. Although HLA matching is not currently used in lung allocation due to organ scarcity and urgency of transplant, understanding its impact could improve risk stratification and outcomes. This study aimed to assess the effect of HLA compatibility on lung graft survival using a large multicentre database and to explore whether this relationship varies by transplant indication.

**Methods:**

We conducted a retrospective cohort study of 37 091 lung transplant recipients in the UNOS/OPTN database. Patients were grouped by HLA mismatch (0–2 *versus* 3–6) and stratified by indication. Multivariate Cox regression and Kaplan–Meier analyses assessed adjusted associations with graft survival.

**Results:**

HLA-DR mismatch was associated with increased graft failure risk at 1 year (hazard ratio (HR) 1.38, 95% CI 1.00–1.90; p=0.048) and 5 years (HR 1.20, 95% CI 1.03–1.40; p=0.020). Stratified analyses showed this effect was found exclusively in those with COPD or interstitial pneumonia. In COPD, HLA-DR mismatch significantly increased failure risk at 1 year (HR 2.88, 95% CI 1.34–6.19; p=0.007). In interstitial pneumonia, 5-year graft failure risk also rose with mismatch (HR 1.28, 95% CI 1.03–1.58; p=0.026).

**Conclusions:**

The present study is the first to demonstrate that HLA-DR mismatch adversely affects lung graft survival in an indication-specific manner. This finding supports the development of indication-specific strategies in post-transplant surveillance and immunosuppression.

## Shareable abstract

**This study of 37 091 lung transplant recipients shows HLA-DR mismatch worsens graft survival in COPD and interstitial pneumonia, but not other indications. HLA matching may enable indication-specific risk stratification and tailored post-transplant care.** https://bit.ly/4tiB7kS

## Graphical abstract

**Figure:**
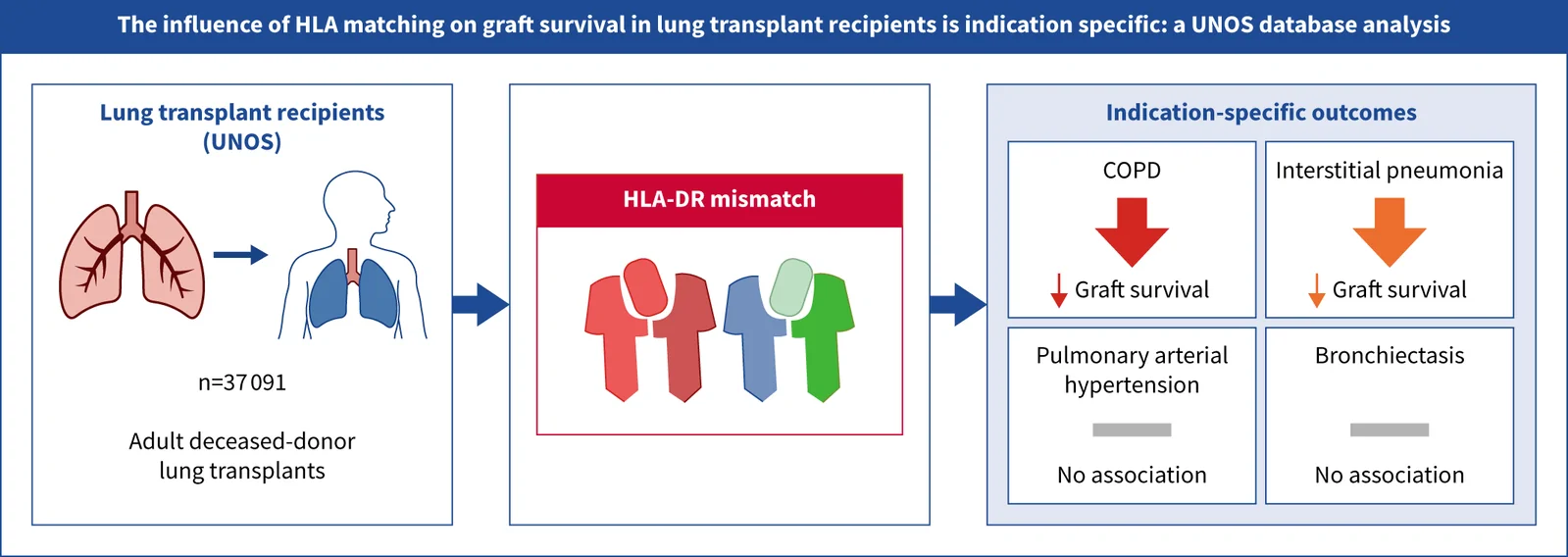
Indication-specific effects of human leukocyte antigen (HLA) mismatch on lung graft survival. In a UNOS (United Network for Organ Sharing) registry analysis of 37 091 lung transplant recipients, HLA-DR mismatch was associated with reduced graft survival at 1 year in COPD and 5 years in interstitial pneumonia, but not in pulmonary arterial hypertension or bronchiectasis.

## Introduction

Lung transplantation is often the only effective treatment option for end-stage pulmonary disease, with over 6000 lung transplants performed worldwide annually [1]. Graft survival after lung transplantation has significantly improved over the past decade, secondary to advancements in organ preservation techniques and better peri- and post-operative management [1, 2]. However, graft survival remains significantly lower than other solid organ transplants, with 5-year lung transplant survival (50%) trailing behind 5-year liver, heart and kidney survival (75%, 85% and 80%, respectively) [3–6].

Antibody-mediated rejection is an important cause of graft failure post-transplant, with antibody production against donor human leukocyte antigens (HLAs) leading to complement cascade activation, endothelial cell injury and inflammation [7]. These donor-specific anti-HLA antibodies (DSAs) are generated in response to HLA mismatch and are associated with a constellation of adverse outcomes including bronchiolitis obliterans syndrome, chronic lung allograft dysfunction, graft loss and patient death [8, 9].

Unlike other solid organ transplants such as kidney, HLA matching is not required for lung transplant allocation, despite evidence suggesting that HLA matching improves clinical outcomes including graft survival, risk of acute rejection and chronic lung allograft dysfunction [8–17]. This is because lung transplant patients are typically critically unwell and therefore cannot wait for a fully, or even partially, matched donor.

A recent study in the liver literature has revealed that the impact of HLA matching on liver graft survival was indication specific, finding a statistically significant association between HLA compatibility and graft survival in patients transplanted for hepatitis, metabolic, drug-induced and congenital indications, but not in patients transplanted for cancer, non-alcoholic steatohepatitis (NASH) or alcoholic cirrhosis. This suggests that the underlying disease process influences the impact that HLA compatibility has on graft survival.

We hypothesise that a similar indication specificity exists within the lung transplant population and that this varying susceptibility to the adverse consequences of HLA matching could be used to risk stratify patients using disease aetiology. This would enable more bespoke care, with more intense surveillance of higher risk groups facilitating earlier and more aggressive intervention. We explored this hypothesis through a large-scale registry analysis, evaluating data from patients registered in the Organ Procurement and Transplant Network (OPTN) Standard Transplant Analysis and Research (STAR) database administered by the United Network for Organ Sharing (UNOS).

## Materials and methods

### Study design and patient selection

This retrospective observational analysis was performed using data for adult lung transplant recipients between 4 October 1999 and 5 August 2022 extracted from the UNOS database (https://unos.org/data). UNOS is a non-profit, private organisation that manages the US organ transplant database, containing all organ transplant data for every transplant event that occurs in the country. We extracted baseline donor characteristics including age, gender, race, body mass index (BMI), donor status, smoking history (>20 pack-years), cytomegalovirus (CMV) and hepatitis C virus (HCV) status, as well as recipient age, gender, race, BMI, blood type, creatinine, forced expiratory volume in 1 s (FEV_1_), forced vital capacity (FVC), insurance type, education level, ischaemic time, and HLA mismatch at HLA-A, HLA-B and HLA-DR.

Inclusion criteria included adult lung transplant recipients receiving a graft from a deceased donor. Patients who have had previous transplants, multi-organ recipients and those receiving a living-donor lung transplant were excluded from the study (n=1845). Patients with implausible data points in variables of interest were excluded from the study (n=1308).

### Exposure and outcome

The primary explanatory variable of interest was HLA mismatch at HLA-A, HLA-B and HLA-DR. The primary outcome was graft survival at 5 years. Secondary, exploratory outcomes were graft survival at 1 and 10 years. Both the primary explanatory variable and primary outcome were defined *a priori*.

### Statistical analysis

Of the 37 091 patients eligible for this study, 27 410 recipients had a complete set of covariates. We performed multiple imputation using chained equations to impute missing data, based on the assumption that data were missing at random. We optimised and assessed the validity of imputed data using convergence curves, strip plots and histograms. We performed complete case analyses sensitivity tests (supplementary table S1) to ensure robustness of results. Patient characteristics were described using mean with standard deviation if continuous and parametric, median with interquartile range if non-parametric, and count with percentage if categorical. Baseline characteristics were compared between patients based on HLA mismatch status, using the t-test or Wilcoxon rank sum test, as appropriate, for continuous variables, and the Chi-squared test for categorical or discrete variables. We stratified patients by indication for transplant, dividing them into patients with COPD, pulmonary arterial hypertension (PAH), bronchiectasis, interstitial pneumonia (including idiopathic pulmonary fibrosis (IPF), sarcoidosis, rheumatoid disease, occupational lung disease, scleroderma, CREST (calcinosis, Raynaud's phenomenon, oesophageal dysmotility, sclerodactyly and telangiectasia) syndrome, pulmonary telangiectasia, and idiopathic interstitial pneumonias such as non-specific interstitial pneumonia, respiratory bronchiolitis, desquamative interstitial pneumonia and acute interstitial pneumonia) and other (including bronchopulmonary dysplasia, congenital malformations, tuberous sclerosis, granulomatous lung disease, tracheopathia osteoplastica, MacLeod syndrome, bronchioloalveolar carcinoma, carcinoid tumourlets and teratoma).

Sample size calculations were conducted using the pwr and effsize R packages to ensure adequate sample size in each HLA category in each indication to obtain a power of at least 0.8 with a significance level of p<0.05 and assuming an effect size of 0.1. Additionally, due to the large number of statistical tests performed in this analysis, we performed a Bonferroni correction, which becomes increasingly conservative with an increasing number of comparisons [18], to ensure the analysis was not biased towards false positives. The primary outcome of interest of graft survival at 5 years was evaluated across four predefined transplant indications (COPD, interstitial pneumonia, bronchiectasis and PAH). Accordingly, the Bonferroni-adjusted significance threshold was set at p=0.0125 (0.05/4). Secondary outcomes of graft survival at 1 and 10 years were considered exploratory and hypothesis generating.

For the primary analysis of graft survival, we first performed univariate Cox proportional regression tests, Kaplan–Meier methods and log-rank tests where the explanatory variable of interest was the number of HLA mismatches both overall and at HLA-A, HLA-B and HLA-DR. These represent the only three HLA loci encoded in the UNOS database. We next developed multivariable proportional hazard models to account for potential confounders. Variables that were significant (p<0.05) on univariate analysis or with a strong clinical or biological relationship with the outcome were included in multivariate analysis model. To ensure model validity, we assessed multicollinearity using the variance inflation factor (VIF) excluding variables with VIF >5. We considered the following variables for multivariate adjustment: recipient education level, FVC, FEV_1_, BMI, creatinine, age, gender, blood group, race, donor CMV status, HCV status, age, gender, smoking history, ischaemic time, procedure type (single *versus* double lung transplant), indication for transplant and year of transplant.

When designing the Cox proportional regression models, we dichotomised overall HLA mismatch (0–2 mismatches=0, 3–6 mismatches=1) to form a categorical variable, consistent with previous database studies exploring the relationship between HLA matching and transplant outcomes [14, 17]. We processed race, transplant year, education level, insurance type, transplant year and indication for transplant as dummy variables. We used variate p-values and Akaike Information Criteria to optimise our model fit, and the Schoenfeld residuals test to ensure the proportional hazards assumption was met. Diagnostic tests were performed to confirm model fit.

All statistical analyses were performed using R version 4.3.1 (R Core Team, Vienna, Austria). Additional packages used were dplyr version 1.0.7 for data manipulation, survminer version 0.4.9 for survival analysis visualisation, mice version 3.14.0 for multiple imputation, transplantr version 0.1.0 for transplant-related functions and mitools version 2.4 for handling multiple imputed datasets (https://cran.r-project.org/web/packages).

## Results

### Patient characteristics

Of the 37 091 patients included in our study 1397 (3.7%) had 0–2 mismatches and 35 694 (96.3%) had 3–6 mismatches. Table 1 summarises patient demographics and characteristics, stratified by total HLA mismatch level. The distribution of indications was similar between the two groups, except for a higher frequency of interstitial pneumonia in the 3–6 HLA mismatches group and a higher frequency of COPD in the 0–2 HLA mismatches group. The two groups were similar in recipient and donor gender, FVC, insurance type, education level, donor age, donor history of smoking, donor circulatory death (DCD) status, ischaemic time, BMI, FEV_1_, transplant procedure type, and transplant year. However, there were notable differences in race distribution, with a higher proportion of White recipients and donors in the 0–2 HLA mismatches group, while the 3–6 HLA mismatches group had a greater proportion of Black recipients and donors.

**TABLE 1:** Patient demographics and characteristics based on total human leukocyte antigen (HLA) mismatch 0–2 *versus* 3–6

	HLA matching	
	0–2 HLA mismatches (n=1397)	3–6 HLA mismatches (n=35 694)
**Recipient factors**		
Age (years)	58 (50–64)	58 (48–64)
BMI (kg·m^−2^)	24.8 (20.9–28.2)	24.9 (21.3–28.5)
Gender		
Male	794 (56.8)	20** **503 (57.4)
Female	603 (43.2)	15** **191 (42.6)
Race		
White	1256 (89.9)	29** **769 (83.4)
Black	56 (4)	3032 (8.5)
Asian	16 (1.1)	537 (1.5)
Other	69 (4.9)	2356 (6.6)
Creatinine (mg·mL^−1^)	0.8 (0.67–1)	0.8 (0.7–1)
FEV_1_ % pred	31 (20–51)	33 (20–52)
FVC % pred	47 (36–60)	47 (37–60)
Blood group		
A	608 (43.5)	14** **307 (40.1)
AB	55 (3.9)	1393 (3.9)
B	128 (9.2)	3992 (11.2)
O	606 (43.4)	16** **002 (44.8)
Insurance		
Private	747 (53.5)	18** **994 (53.2)
Government	618 (44.2)	16** **072 (45)
Other	32 (2.3)	628 (1.8)
Education level		
No high school	0 (0)	55 (0.2)
High school	47 (3.4)	1085 (3)
Some college	604 (43.2)	14** **481 (40.6)
College degree or higher	439 (31.4)	20** **073 (56.2)
**Donor factors**		
Age (years)	33 (22–45)	32 (22–45)
Gender		
Male	861 (61.6)	21** **737 (60.9)
Female	536 (38.4)	13** **957 (39.1)
Race		
White	1136 (81.3)	22** **959 (64.3)
Black	93 (6.7)	6458 (18.1)
Asian	20 (1.4)	924 (2.6)
Other	148 (10.6)	5353 (15)
DCD	28 (2)	797 (2.2)
Donor anti-CMV positive	797 (57.1)	21** **742 (60.9)
Donor anti-HCV positive	14 (1)	443 (1.2)
Donor smoker	215 (15.4)	5180 (14.5)
**Indications**		
COPD	528 (37.8)	11** **751 (32.9)
Pulmonary arterial hypertension	58 (4.2)	1761 (4.9)
Interstitial pneumonia	607 (43.5)	16** **981 (47.6)
Bronchiectasis	191 (13.7)	4906 (13.7)
Other	13 (0.9)	295 (0.8)
**Transplant factors**		
Ischaemic time (min)	294 (230–364)	296 (233–363)
Procedure		
Single lung	517 (37)	13** **107 (36.7)
Double lung	880 (63)	22** **587 (63.3)
Transplant year		
2010–present	788 (56.4)	20** **341 (57)
2000–2009	420 (30.1)	10** **834 (30.4)
1980–1999	189 (13.5)	4519 (12.6)

Data are presented as median (interquartile range) or n (%). BMI: body mass index; FEV_1_: forced expiratory volume in 1 s; FVC: forced vital capacity; DCD: donor circulatory death; CMV: cytomegalovirus; HCV: hepatitis C virus.

### Univariate and Kaplan–Meier survival analysis

Univariate Cox proportional hazards analysis found a statistically significant increase in risk of graft loss in the 3–6 HLA mismatches group *versus* the 0–2 HLA mismatches group at 5 years (hazard ratio (HR) 1.29, 95% CI 1.09–1.52; p=0.003) and 10 years (HR 1.27, 95% CI 1.10–1.46; p<0.001) (table 2).

**TABLE 2:** Univariate Cox survival analysis

	1 years		5 years		10 years	
	HR (95% CI)	p-value	HR (95% CI)	p-value	HR (95% CI)	p-value
**Recipient factors**						
Age (years)	1.00 (0.99–1.00)	0.205	1.00 (1.00–1.00)	0.043	1.01 (1.01–1.01)	<0.001
BMI (kg·m^−2^)	1.02 (1.01–1.04)	<0.001	1.01 (1.01–1.02)	<0.001	1.02 (1.02–1.03)	<0.001
Gender (male=1, female=0)	0.98 (0.89–1.09)	0.751	0.97 (0.91–1.02)	0.208	0.93 (0.88–0.97)	0.001
Race: White	Reference		Reference		Reference	
Race: Black	1.19 (1.01–1.40)	0.042	1.21 (1.10–1.33)	<0.001	1.18 (1.09–1.28)	<0.001
Race: Asian	0.91 (0.59–1.41)	0.675	1.01 (0.79–1.30)	0.94	1.14 (0.92–1.42)	0.219
Race: Other	1.08 (0.88–1.31)	0.464	1.23 (1.10–1.37)	<0.001	1.29 (1.17–1.43)	<0.001
Creatinine (mg·mL^−1^)	1.05 (1.01–1.09)	0.016	1.01 (0.96–1.05)	0.802	0.97 (0.91–1.02)	0.238
FEV_1_ % pred	1.00 (0.99–1.00)	0.035	1.00 (1.00–1.00)	<0.001	1.00 (1.00–1.00)	<0.001
FVC % pred	1.00 (1.00–1.01)	0.001	1.00 (1.00–1.00)	<0.001	1.00 (1.00–1.00)	<0.001
Blood group: A	Reference		Reference		Reference	
Blood group: AB	1.20 (0.95–1.53)	0.13	1.13 (0.99–1.30)	0.071	1.11 (0.98–1.25)	0.097
Blood group: B	0.96 (0.81–1.13)	0.61	1.07 (0.97–1.17)	0.169	1.03 (0.95–1.11)	0.523
Blood group: O	1.05 (0.94–1.17)	0.377	1.04 (0.98–1.11)	0.171	1.06 (1.01–1.12)	0.019
Insurance: Private	Reference		Reference		Reference	
Insurance: Government	0.97 (0.88–1.07)	0.555	1.24 (1.18–1.31)	<0.001	1.36 (1.30–1.43)	<0.001
Insurance: Other	0.68 (0.43–1.09)	0.107	0.73 (0.57–0.93)	0.012	0.65 (0.53–0.80)	<0.001
Education level: No high school	Reference		Reference		Reference	
Education level: High school	0.89 (0.21–3.70)	0.874	1.21 (0.50–2.90)	0.676	1.37 (0.67–2.82)	0.387
Education level: Some college	0.90 (0.22–3.63)	0.881	1.13 (0.48–2.65)	0.784	1.18 (0.58–2.41)	0.64
Education level: College degree or higher	0.87 (0.22–3.52)	0.847	1.14 (0.49–2.70)	0.757	1.23 (0.61–2.50)	0.564
**Donor factors**						
Age (years)	1.01 (1.01–1.01)	<0.001	1.01 (1.01–1.01)	<0.001	1.01 (1.01–1.01)	<0.001
Gender (male=1, female=0)	1.21 (1.09–1.33)	<0.001	1.02 (0.96–1.08)	0.494	1.02 (0.97–1.07)	0.377
Race: White	Reference		Reference		Reference	
Race: Black	1.24 (1.10–1.41)	0.001	1.24 (1.16–1.34)	<0.001	1.33 (1.25–1.41)	<0.001
Race: Asian	1.22 (0.92–1.63)	0.173	1.22 (1.03–1.44)	0.024	1.24 (1.07–1.44)	0.004
Race: Other	0.98 (0.85–1.14)	0.812	1.11 (1.02–1.20)	0.014	1.15 (1.07–1.23)	<0.001
DCD (no=0, yes=1)	1.40 (1.02–1.90)	0.035	1.82 (1.49–2.22)	<0.001	1.82 (1.50–2.20)	<0.001
Donor anti-CMV (negative=0, positive=1)	1.07 (0.97–1.18)	0.176	1.18 (1.11–1.25)	<0.001	1.19 (1.13–1.24)	<0.001
Donor anti-HCV (negative=0, positive=1)	1.18 (0.68–2.03)	0.555	3.95 (2.66–5.86)	<0.001	2.75 (1.85–4.09)	<0.001
Smoker (no=0, yes=1)	1.25 (1.10–1.43)	0.001	0.96 (0.89–1.03)	0.249	0.85 (0.80–0.91)	<0.001
**Indication for transplant**						
COPD	Reference		Reference		Reference	
Pulmonary arterial hypertension	2.27 (1.86–2.78)	<0.001	1.54 (1.36–1.75)	<0.001	1.17 (1.04–1.31)	0.006
Interstitial pneumonia	1.55 (1.38–1.74)	<0.001	1.38 (1.30–1.48)	<0.001	1.42 (1.34–1.50)	<0.001
Bronchiectasis	1.08 (0.91–1.28)	0.36	1.29 (1.19–1.40)	<0.001	1.03 (0.96–1.11)	0.379
**Transplant factors**						
Ischaemic time (min)	1.00 (1.00–1.00)	<0.001	1.00 (1.00–1.00)	<0.001	1.00 (1.00–1.00)	0.599
Procedure type (single lung=0, double=1)	0.83 (0.75–0.92)	<0.001	1.00 (0.94–1.05)	0.907	0.90 (0.86–0.94)	<0.001
HLA-A (2 mismatches *versus* 0 reference)	0.99 (0.81–1.21)	0.936	1.14 (1.02–1.29)	0.024	1.13 (1.02–1.24)	0.018
HLA-B (2 mismatches *versus* 0 reference)	0.87 (0.62–1.21)	0.41	1.16 (0.94–1.43)	0.18	1.10 (0.92–1.31)	0.294
HLA-DR (2 mismatches *versus* 0 reference)	1.42 (1.06–1.88)	0.017	1.20 (1.05–1.38)	0.007	1.23 (1.09–1.40)	0.001
**Overall**						
HLA mismatch (0–2 *versus* 3–6 mismatches)	1.21 (0.90–1.67)	0.20	1.29 (1.09–1.52)	0.003	1.27 (1.10–1.46)	<0.001

HR: hazard ratio; BMI: body mass index; FEV_1_: forced expiratory volume in 1 s; FVC: forced vital capacity; DCD: donor circulatory death; CMV: cytomegalovirus; HCV: hepatitis C virus; HLA: human leukocyte antigen.

Further analysis using univariate Cox models identified a significantly higher graft loss hazard with HLA-DR mismatch at 1 year (HR 1.42, 95% CI 1.06–1.88; p=0.017), 5 years (HR 1.20, 95% CI 1.05–1.38; p=0.007) and 10 years (HR 1.23, 95% CI 1.09–1.40; p=0.001), and with HLA-A mismatch at 5 years (HR 1.14, 95% CI 1.02–1.29; p=0.024) and 10 years (HR 1.13, 95% CI 1.02–1.24; p=0.018). There was no association with HLA-B mismatch and graft survival at 1, 5 or 10 years.

**FIGURE 1:**
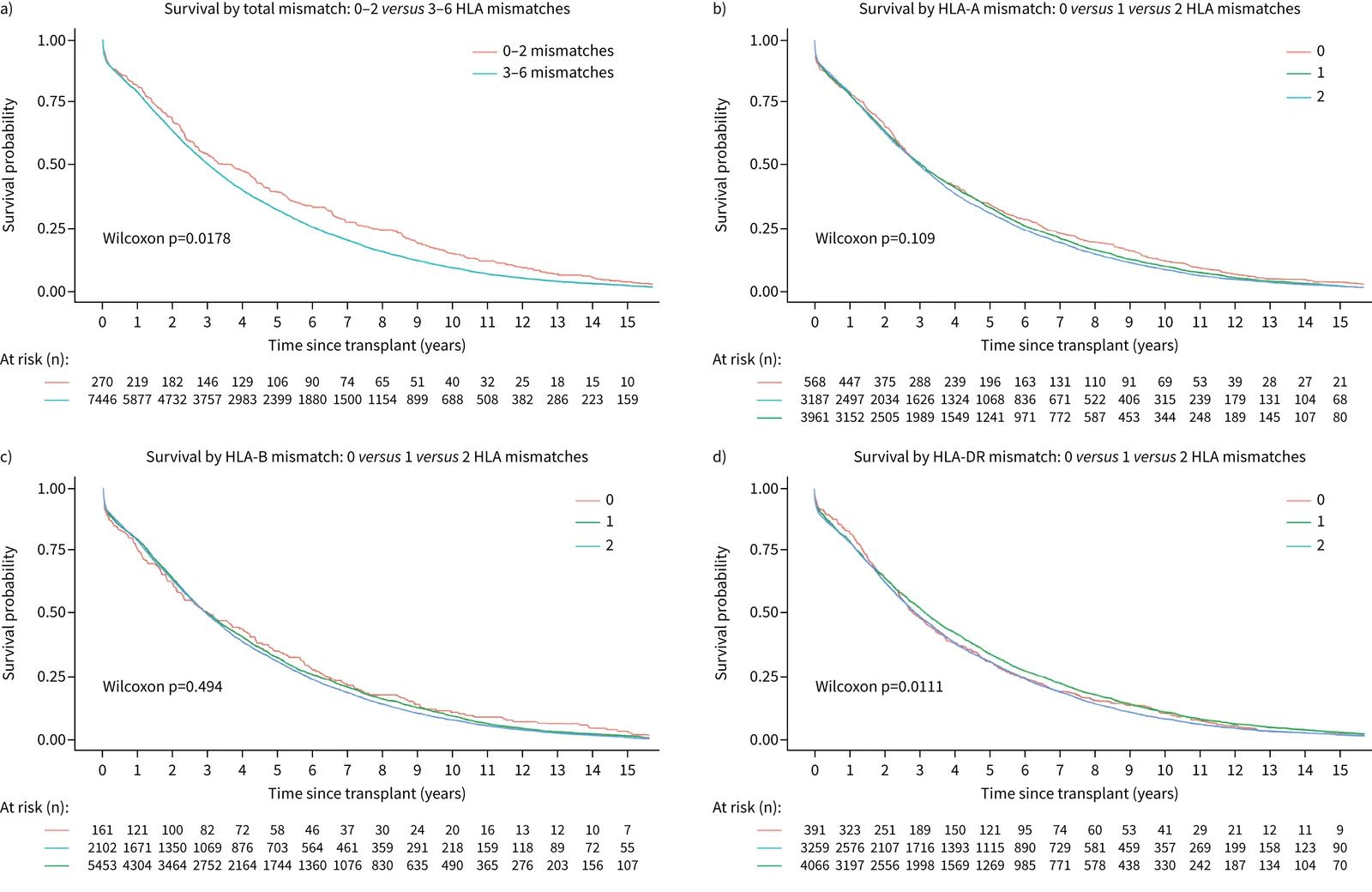
Kaplan–Meier survival functions of time to graft loss by a) total human leukocyte antigen (HLA) mismatch 0–2 *versus* 3–6 mismatches, b) HLA-A locus mismatch 0 *versus* 1 *versus* 2 mismatches, c) HLA-B locus mismatch 0 *versus* 1 *versus* 2 mismatches and d) HLA-DR locus mismatch 0 *versus* 1 *versus* 2 mismatches.

The Kaplan–Meier survival function curves for overall mismatch, HLA-A mismatch, HLA-B mismatch and HLA-DR mismatch are presented in figure 1a–d, respectively, and are consistent with the univariate Cox models.

Several covariates were found to be significant in the univariate Cox analysis across all three time-points, including elevated recipient BMI, donor age, DCD status, Black donor and recipient race, as well as PAH and interstitial pneumonia transplant indication. Due to their potential confounding effect on graft survival, these covariates were included in the multivariate models.

When we split our cohort by indication (COPD, interstitial pneumonia, bronchiectasis and PAH), clear inter-indication variability emerged regarding the impact of HLA mismatch on graft survival (table 3). In COPD, overall HLA mismatch at 1 year (HR 2.46, 95% CI 1.08–5.59; p=0.031), 5 years (HR 1.54, 95% CI 1.14–2.07; p=0.004) and 10 years (HR 1.38, 95% CI 1.10–1.73; p=0.005) was associated with increased graft loss hazard. In PAH, interstitial pneumonia and bronchiectasis there was no statistically significant association between overall HLA mismatch and graft survival.

**TABLE 3:** Univariate Cox survival analysis stratified by indication

	1 years		5 years		10 years	
	HR (95% CI)	p-value	HR (95% CI)	p-value	HR (95% CI)	p-value
**COPD**						
Overall mismatch (0–2 *versus* 3–6 mismatches)	2.46 (1.08–5.59)	0.031	1.54 (1.14–2.07)	0.004	1.38 (1.10–1.73)	0.005
HLA-A (2 mismatches *versus* 0 reference)	0.88 (0.60–1.28)	0.495	1.08 (0.88–1.33)	0.435	1.07 (0.91–1.27)	0.391
HLA-B (2 mismatches *versus* 0 reference)	1.03 (0.50–2.12)	0.931	1.39 (0.94–2.04)	0.097	1.11 (0.82–1.52)	0.493
HLA-DR (2 mismatches *versus* 0 reference)	2.85 (1.33–6.13)	0.007	1.20 (0.94–1.53)	0.152	1.24 (1.01–1.52)	0.039
**Interstitial pneumonia**						
Overall mismatch (0–2 *versus* 3–6 mismatches)	0.96 (0.66–1.39)	0.822	1.24 (0.97–1.59)	0.087	1.19 (0.95–1.48)	0.124
HLA-A (2 mismatches *versus* 0 reference)	1.05 (0.79–1.39)	0.73	1.22 (1.02–1.46)	0.03	1.10 (0.94–1.30)	0.247
HLA-B (2 mismatches *versus* 0 reference)	0.71 (0.46–1.10)	0.123	1.02 (0.74–1.41)	0.903	1.02 (0.76–1.36)	0.899
HLA-DR (2 mismatches *versus* 0 reference)	1.10 (0.77–1.57)	0.615	1.31 (1.06–1.62)	0.012	1.31 (1.07–1.60)	0.008
**Bronchiectasis**						
Overall mismatch (0–2 *versus* 3–6 mismatches)	0.98 (0.46–2.09)	0.967	0.94 (0.67–1.32)	0.706	1.08 (0.80–1.44)	0.622
HLA-A (2 mismatches *versus* 0 reference)	1.12 (0.58–2.16)	0.727	1.07 (0.82–1.39)	0.625	1.14 (0.91–1.43)	0.244
HLA-B (2 mismatches *versus* 0 reference)	0.89 (0.33–2.40)	0.824	1.01 (0.61–1.68)	0.969	1.08 (0.72–1.62)	0.712
HLA-DR (2 mismatches *versus* 0 reference)	1.40 (0.66–2.97)	0.379	0.99 (0.74–1.34)	0.971	1.04 (0.79–1.36)	0.795
**Pulmonary arterial hypertension**						
Overall mismatch (0–2 *versus* 3–6 mismatches)	1.08 (0.34–3.42)	0.897	1.36 (0.56–3.32)	0.501	1.34 (0.62–2.91)	0.458
HLA-A (2 mismatches *versus* 0 reference)	0.90 (0.44–1.85)	0.773	1.01 (0.62–1.66)	0.958	1.13 (0.71–1.80)	0.619
HLA-B (2 mismatches *versus* 0 reference)	1.54 (0.24–10.10)	0.651	1.17 (0.46–2.94)	0.74	1.31 (0.62–2.79)	0.478
HLA-DR (2 mismatches *versus* 0 reference)	1.18 (0.50–2.77)	0.706	1.07 (0.65–1.77)	0.794	1.13 (0.70–1.83)	0.62

HR: hazard ratio; HLA: human leukocyte antigen.

Regarding HLA mismatch at HLA-A, HLA-B and HLA-DR in COPD, HLA-DR mismatch at both 1 year (HR 2.85, 95% CI 1.32–6.13; p=0.007) and 10 years (HR 1.24, 95% CI 1.01–1.52; p=0.039) was associated with increased risk of graft loss (table 3). In interstitial pneumonia, HLA-DR mismatch at 5 years (HR 1.31, 95% CI 1.06–1.62; p=0.012) and 10 years (HR 1.31, 95% CI 1.07–1.60; p=0.008) was associated with increased graft loss hazard, as was HLA-A mismatch at 5 years (HR 1.22, 95% CI 1.02–1.46; p=0.03). One mismatch at HLA-B in the interstitial pneumonia cohort was associated with increased graft survival (HR 0.64, 95% CI 0.4–0.99; p=0.045). In both bronchiectasis and PAH there was no association between graft survival and mismatch at HLA-A, HLA-B or HLA-DR.

### Multivariate survival analysis

When we adjusted for covariates using multivariate Cox proportional hazards analysis, the relationship between overall HLA mismatch and graft survival was lost (table 4). The relationship between HLA-DR mismatch and reduced graft survival, however, was preserved, with HLA-DR mismatch leading to an increased graft failure hazard at 1 year (HR 1.38, 95% CI 1.00–1.90; p=0.048) and 5 years (HR 1.20, 95% CI 1.03–1.40; p=0.02).

**TABLE 4:** Multivariate Cox survival analysis

	1 years		5 years		10 years	
	HR (95% CI)	p-value	HR (95% CI)	p-value	HR (95% CI)	p-value
**Recipient factors**						
Age (years)	1.0 (0.99–1.0)	0.17	0.99 (0.99–0.99)	<0.001	1.0 (1.0–1.0)	0.16
BMI (kg·m^−2^)	1.02 (1.01–1.03)	<0.01	1.01 (1.0–1.02)	0.01	1.01 (1.0–1.02)	<0.01
Gender (male=1, female=0)	0.97 (0.86–1.08)	0.54	1.01 (0.95–1.07)	0.74	0.99 (0.94–1.05)	0.79
Race: White	Reference		Reference		Reference	
Race: Black	1.03 (0.86–1.22)	0.77	1.06 (0.96–1.17)	0.23	1.04 (0.95–1.13)	0.41
Race: Asian	0.8 (0.51–1.25)	0.33	0.88 (0.68–1.14)	0.34	0.96 (0.77–1.19)	0.68
Race: Other	0.92 (0.75–1.14)	0.46	1.03 (0.92–1.16)	0.6	1.02 (0.92–1.13)	0.74
Creatinine (mg·mL^−1^)	1.03 (0.99–1.08)	0.17	1.01 (0.98–1.05)	0.48	1.03 (0.98–1.09)	0.19
FEV_1_ % pred	1.0 (0.99–1.0)	0.11	1.0 (1.0–1.0)	0.68	1.0 (1.0–1.0)	0.54
FVC % pred	1.0 (0.99–1.0)	0.3	1.0 (1.0–1.0)	0.06	1.0 (1.0–1.0)	0.69
Blood group: A	Reference		Reference		Reference	
Blood group: AB	1.19 (0.94–1.51)	0.15	1.13 (0.98–1.29)	0.09	1.11 (0.99–1.25)	0.08
Blood group: B	0.9 (0.76–1.08)	0.26	1.03 (0.94–1.13)	0.53	1.0 (0.92–1.09)	0.93
Blood group: O	1.03 (0.92–1.14)	0.63	1.0 (0.94–1.06)	0.92	1.02 (0.97–1.07)	0.53
Insurance: Private	Reference					
Insurance: Government	1.06 (0.96–1.18)	0.25	1.21 (1.14–1.28)	<0.001	1.11 (1.06–1.17)	<0.001
Insurance: Other	0.61 (0.38–0.98)	0.04	0.81 (0.63–1.04)	0.1	0.81 (0.66–1.0)	0.05
Education level: No high school	Reference		Reference		Reference	
Education level: High school	0.88 (0.21–3.68)	0.86	1.25 (0.52–3.02)	0.62	1.03 (0.5–2.13)	0.94
Education level: Some college	0.88 (0.22–3.61)	0.86	1.17 (0.5–2.75)	0.72	0.95 (0.46–1.93)	0.88
Education level: College degree or higher	0.84 (0.21–3.44)	0.81	1.14 (0.49–2.69)	0.76	0.95 (0.46–1.93)	0.88
**Donor factors**						
Age (years)	1.01 (1.0–1.01)	<0.001	1.01 (1.01–1.01)	<0.001	1.0 (1.0–1.01)	<0.001
Gender (male=1, female=0)	1.12 (1.0–1.25)	0.05	0.93 (0.87–0.99)	0.02	0.98 (0.93–1.03)	0.43
Race: White	Reference		Reference		Reference	
Race: Black	1.32 (1.16–1.5)	<0.001	1.14 (1.06–1.23)	<0.001	1.12 (1.05–1.2)	<0.001
Race: Asian	1.23 (0.92–1.66)	0.16	1.07 (0.9–1.27)	0.43	0.99 (0.85–1.15)	0.92
Race: Other	1.06 (0.91–1.23)	0.48	1.03 (0.95–1.12)	0.45	1.03 (0.96–1.11)	0.4
DCD (no=0, yes=1)	1.42 (1.04–1.96)	0.03	1.62 (1.32–1.98)	<0.001	1.4 (1.15–1.7)	<0.001
Donor anti-CMV (negative=0, positive=1)	1.03 (0.93–1.15)	0.54	1.12 (1.05–1.19)	<0.001	1.06 (1.01–1.12)	0.02
Donor anti-HCV (negative=0, positive=1)	1.22 (0.7–2.1)	0.48	3.78 (2.56–5.58)	<0.001	2.37 (1.63–3.44)	<0.001
Smoker (no=0, yes=1)	1.12 (0.98–1.29)	0.11	1.06 (0.98–1.14)	0.18	1.08 (1.01–1.16)	0.02
**Indication for transplant**						
COPD	Reference					
Pulmonary arterial hypertension	2.81 (2.16–3.64)	<0.001	1.52 (1.3–1.78)	<0.001	1.27 (1.1–1.45)	<0.001
Interstitial pneumonia	1.76 (1.47–2.11)	<0.001	1.21 (1.1–1.34)	<0.001	1.11 (1.02–1.21)	0.01
Bronchiectasis	1.17 (0.92–1.47)	0.2	1.17 (1.03–1.32)	0.01	1.1 (0.99–1.22)	0.07
**Transplant factors**						
Ischaemic time (min)	1.0 (1.0–1.0)	<0.001	1.0 (1.0–1.0)	0.02	1.0 (1.0–1.0)	0.99
Procedure type (single lung=0, double=1)	0.76 (0.67–0.85)	<0.001	0.82 (0.76–0.87)	<0.001	0.81 (0.76–0.86)	<0.001
HLA-A (1 mismatch *versus* 0 reference)	0.97 (0.78–1.2)	0.78	1.03 (0.91–1.17)	0.62	1.01 (0.91–1.13)	0.81
HLA-A (2 mismatches *versus* 0 reference)	0.94 (0.74–1.19)	0.58	1.12 (0.98–1.29)	0.1	1.06 (0.95–1.2)	0.3
HLA-B (1 mismatch *versus* 0 reference)	0.82 (0.58–1.17)	0.28	1.04 (0.83–1.29)	0.76	0.98 (0.81–1.19)	0.84
HLA-B (2 mismatches *versus* 0 reference)	0.82 (0.57–1.19)	0.3	1.08 (0.86–1.37)	0.5	1.0 (0.82–1.22)	0.99
HLA-DR (1 mismatch *versus* 0 reference)	1.34 (0.99–1.81)	0.06	1.11 (0.96–1.29)	0.15	1.05 (0.92–1.2)	0.49
HLA-DR (2 mismatches *versus* 0 reference)	1.38 (1.0–1.9)	0.05	1.2 (1.03–1.4)	0.02	1.1 (0.95–1.28)	0.19
Overall HLA mismatch (0–2 *versus* 3–6 mismatches)	1.16 (0.8–1.68)	0.43	1.1 (0.89–1.36)	0.09	1.15 (0.97–1.36)	0.14

HR: hazard ratio; BMI: body mass index; FEV_1_: forced expiratory volume in 1 s; FVC: forced vital capacity; DCD: donor circulatory death; CMV: cytomegalovirus; HCV: hepatitis C virus; HLA: human leukocyte antigen.

As in univariate analysis, there was clear inter-indication variability in the influence of HLA mismatch on graft survival (table 5). Regarding overall mismatch, the association between HLA mismatch and graft loss was maintained in COPD at 1 year (HR 2.39, 95% CI 1.05–5.44; p=0.037), 5 years (HR 1.49, 95% CI 1.11–2.01; p=0.008) and 10 years (HR 1.36, 95% CI 1.08–1.70; p=0.008). As observed in univariate analysis, in PAH, interstitial pneumonia and bronchiectasis there was no statistically significant association between overall HLA mismatch and graft survival.

**TABLE 5:** Multivariate Cox survival analysis stratified by indication

	1 years		5 years		10 years	
	HR (95% CI)	p-value	HR (95% CI)	p-value	HR (95% CI)	p-value
**COPD**						
Overall mismatch (0–2 *versus* 3–6 mismatches)	2.39 (1.05–5.44)	0.037	1.49 (1.11–2.01)	0.008	1.36 (1.08–1.70)	0.008
HLA-A (2 mismatches *versus* 0 reference)	0.86 (0.59–1.25)	0.426	1.03 (0.84–1.27)	0.762	1.06 (0.9–1.25)	0.469
HLA-B (2 mismatches *versus* 0 reference)	0.96 (0.46–1.97)	0.903	1.33 (0.9–1.97)	0.149	1.15 (0.84–1.58)	0.368
HLA-DR (2 mismatches *versus* 0 reference)	2.88 (1.34–6.19)	0.007	1.14 (0.89–1.47)	0.305	1.08 (0.88–1.33)	0.443
**Interstitial pneumonia**						
Overall mismatch (0–2 *versus* 3–6 mismatches)	0.94 (0.65–1.36)	0.740	1.21 (0.94–1.55)	0.133	1.14 (0.92–1.42)	0.236
HLA-A (2 mismatches *versus* 0 reference)	1.0 (0.75–1.33)	0.998	1.16 (0.97–1.39)	0.107	1.04 (0.88–1.22)	0.671
HLA-B (2 mismatches *versus* 0 reference)	0.73 (0.47–1.12)	0.149	0.94 (0.68–1.31)	0.721	0.87 (0.64–1.17)	0.347
HLA-DR (2 mismatches *versus* 0 reference)	1.08 (0.75–1.54)	0.686	1.28 (1.03–1.58)	0.026	1.19 (0.98–1.46)	0.083
**Bronchiectasis**						
Overall mismatch (0–2 *versus* 3–6 mismatches)	1.00 (0.47–2.13)	0.99	0.87 (0.62–1.22)	0.409	0.96 (0.71–1.29)	0.771
HLA-A (2 mismatches *versus* 0 reference)	1.06 (0.55–2.07)	0.854	1.05 (0.8–1.37)	0.744	1.13 (0.89–1.43)	0.317
HLA-B (2 mismatches *versus* 0 reference)	0.85 (0.31–2.32)	0.746	0.89 (0.53–1.48)	0.646	0.89 (0.59–1.33)	0.57
HLA-DR (2 mismatches *versus* 0 reference)	1.5 (0.7–3.18)	0.296	0.93 (0.69–1.25)	0.616	0.94 (0.72–1.23)	0.665
**Pulmonary arterial hypertension**						
Overall mismatch (0–2 *versus* 3–6 mismatches)	1.00 (0.31–3.29)	0.99	1.53 (0.62–3.79)	0.3697043	1.36 (0.61–3.07)	0.454
HLA-A (2 mismatches *versus* 0 reference)	0.88 (0.42–1.84)	0.729	1.05 (0.63–1.75)	0.851	1.14 (0.71–1.83)	0.585
HLA-B (2 mismatches *versus* 0 reference)	1.62 (0.23–11.21)	0.625	1.23 (0.48–3.19)	0.666	1.23 (0.55–2.72)	0.616
HLA-DR (2 mismatches *versus* 0 reference)	1.34 (0.55–3.27)	0.524	1.03 (0.61–1.75)	0.9	1.28 (0.75–2.16)	0.363

HR: hazard ratio; HLA: human leukocyte antigen.

Regarding HLA locus matching, in PAH and bronchiectasis there was no association between graft survival and overall mismatch at HLA-A, HLA-B or HLA-DR. In COPD, HLA-DR mismatch was associated with an increased graft failure hazard at 1 year (HR 2.88, 95% CI 1.34–6.19; p=0.007). In interstitial pneumonia, HLA-DR mismatch was associated with an increased graft failure hazard at 5 years (HR 1.28, 95% CI 1.03–1.58; p=0.026). With a corrected significance cut-off of 0.0125 using the Bonferroni correction, only the association between overall HLA mismatch and graft survival and HLA-DR mismatch and graft survival in COPD remains significant.

## Discussion

The present study is the largest to investigate the relationship between HLA matching and lung transplant graft survival. We demonstrated that two mismatches at HLA-DR increased the risk for graft loss in lung transplant recipients at 1 and 5 years. This relationship between HLA-DR mismatch and graft survival was indication specific, seen exclusively in patients with COPD and IP.

The literature surrounding the role of HLA locus matching in lung transplant graft survival is inconsistent, and poor survival has been reported in association with mismatch at the A, B and DR loci [17]. However there is a growing consensus on the importance of HLA-DR matching, with large-scale cohort studies using HLA epitope mismatching algorithms identifying HLA-DR mismatch as a risk factor for graft loss [19], and registry studies using the same (UNOS) database as our work demonstrating a significant association between HLA-DR mismatch and graft survival [15, 16].

The importance of HLA-DR mismatch has been reported in a range of other solid organ transplants. This is especially evident in kidney transplantation, with the USA considering only HLA-DR for allocation points and the UK favouring HLA-DR over HLA-B. While the exact mechanism behind this relationship between HLA-DR mismatch and graft loss is still under investigation, it has been demonstrated that HLA-DR mismatch is correlated with increased production of DSAs [20].

Taking advantage of our large sample size, we were able to show that the effect of HLA-DR mismatch on lung graft survival is only observed in certain indications for lung transplant. We demonstrated that 2 HLA-DR mismatches in patients transplanted for COPD was associated with an increased risk of graft failure at 1 year (HR 2.88, 95% CI 1.34–6.19; p=0.007) and patients transplanted for interstitial pneumonia at 5 years (HR 1.28, 95% CI 1.03–1.58; p=0.026). We did not find a statistically significant relationship between graft survival and mismatch at HLA-A, HLA-B or HLA-DR at 1, 5 or 10 years in transplants for pulmonary hypertension or bronchiectasis.

This indication specificity has been reported in the liver literature, with a recent paper by Nabulsi *et al.* [21] identifying that in liver transplantation, HLA mismatch was associated with reduced graft survival in recipients suffering from hepatitis, metabolic, drug toxicity and congenital indications. This effect was not seen in patients transplanted for cancer, NASH and alcoholic cirrhosis. This was explained using the “dualistic theory”; a theory originating from Markus *et al.* [22] in 1988, which posits that the immune response generated by HLA incompatibility is influenced by the underlying disease aetiology. For example, the negative impact of HLA mismatch on graft survival in autoimmune disease may be attenuated as grafts with greater HLA incompatibility would be seen as further from self, and less prone to autoimmune attack and disease recurrence.

This approach can also be used in the context of our findings, with both interstitial pneumonia and COPD characterised by chronic inflammation, leading to persistent activation of fibroblasts, macrophages and resident T-cells [23, 24]. In contrast, bronchiectasis and PAH are not typically characterised by pervasive systemic immune activation. HLA-DR mismatch is associated with the development of bronchiolitis obliterans syndrome, attributed to the indirect pathway of allorecognition where mismatched HLA-DR antigens presented by recipient antigen-presenting cells activate CD4^+^ T-cells, leading to chronic immune-mediated injury [25]. The chronic inflammation seen in COPD and interstitial pneumonia could accelerate this process, making these indications for transplant more susceptible to chronic immune-mediated injury. This chronic inflammation-based argument is reinforced by the findings of our IPF subgroup analysis, where the relationship between HLA-DR mismatch and graft survival at 5 years was lost. IPF is primarily characterised by aberrant epithelial injury and fibroproliferative repair rather than sustained immune-mediated or inflammatory pathology. The absence of a detectable HLA-DR mismatch effect in this subgroup therefore supports a role for underlying inflammatory or immune activation in mediating the indication-specific influence of HLA mismatch on lung allograft survival. Furthermore, interstitial pneumonia and COPD have been associated with increased expression of HLA-DR in lung tissue, particularly in alveolar macrophages [26–30]. Thus, the allograft not only introduces mismatched antigens but also encounters a chronically active immune system primed for hyper-responsiveness, increasing the risk for graft rejection and loss.

Regarding the clinical implications of our findings, the limited donor pool and urgency of lung transplantation mean that the benefits of HLA matching are not significant enough to justify changes in organ allocation protocols. Lung transplant candidates are typically more critically unwell than recipients of other solid organ transplants and cannot safely remain on the waiting list for prolonged periods while awaiting a better HLA-matched organ. Consistent with this, waiting-list mortality remains substantially higher for lung transplantation (17 deaths per 100 patient-years) than for solid organ transplants where HLA matching is routinely performed [31], such as kidney transplantation (5 deaths per 100 patient-years) [32]. In addition, thoracic organs have significantly shorter acceptable cold ischaemic times, necessitating rapid donor–recipient matching within geographically constrained donor pools. This limits the opportunity for extensive immunological matching beyond blood group compatibility. Consequently, organ allocation in lung transplantation prioritises urgency, blood group compatibility, donor lung size and organ quality over HLA matching. However, as *ex vivo* lung perfusion techniques continue to advance, more nuanced factors influencing graft survival, such as HLA compatibility, may become increasingly relevant for organ allocation in the future. In terms of current clinical application, understanding the role of HLA mismatch, and specifically how this role is influenced by transplant indication, could allow for accurate risk stratification of patients and tailoring of post-transplant care and surveillance. This potential benefits of HLA-specific management have already been documented in kidney transplantation, where Sureshkumar and Chopra [33] have demonstrated that depleting antibody induction therapy with thymoglobulin or alemtuzumab mitigates the negative impact of HLA-DR mismatch on kidney graft survival. A similar approach of using depleting antibody induction therapy in indications where HLA-DR mismatch is known to lead to adverse outcomes, *e.g.* in COPD, could help prolong graft survival and improve patient outcomes.

The present study is limited by the inherent limitations of large-scale, retrospective database studies. The UNOS database contains a significant amount of missing data, and although we used complete case sensitivity analysis to ensure robust imputation of missing data, this still represents a potential limitation if data were missing not at random. Furthermore, we were not able to interrogate potentially relevant outcomes and variables not coded by the UNOS database. For example, we were limited to HLA-A, HLA-B and HLA-DR, and therefore could not explore the effect of other HLA loci such as HLA-DQ which have recently been shown to influence lung transplant graft survival through increasing the risk of chronic lung allograft dysfunction [34]. Additionally, we did not have access to lymphocyte cross-match results or quantitative DSA data. While recipient HLA typing and donor HLA mismatch information were available, and transplant centres routinely perform virtual cross-matching using recipient antibody profiles at the time of organ offer [35], the registry does not capture centre-level virtual cross-match assessments or DSA measurements. As such, we were unable to account for the presence or strength of pre-formed DSA or the results of any retrospective cross-matching performed post-transplant. Likewise, we did not have data on the immunosuppression regimes that lung transplant recipients were subject to, a limitation inherent to the UNOS database and indeed large registry analyses.

Finally, it is important to highlight that PAH and bronchiectasis comprised the smallest indication subgroups in this cohort, representing approximately 5% and 14% of recipients, respectively. As such, analyses within these groups were relatively underpowered compared with more prevalent indications, and the absence of statistically significant associations should be interpreted with caution. In these indications, a lack of observed effect may therefore reflect limited sample size rather than a true absence of association.

In conclusion, the present study is the first to demonstrate that HLA matching influences lung transplant graft survival in an indication-specific fashion, with HLA-DR mismatch increasing risk of graft failure at 1 year in COPD and at 5 years in interstitial pneumonia. Further studies are required to better delineate the molecular mechanisms underlying this indication specificity, facilitating better understanding and management of these different subsections of lung transplant recipients.

## Supplementary material

**Please note:** supplementary material is not edited by the Editorial Office, and is uploaded as it has been supplied by the author. Supplementary material ERJ-01890-2025.Supplement

## References

[1] Khush KK, Cherikh WS, Chambers DC, et al. The International Thoracic Organ Transplant Registry of the International Society for Heart and Lung Transplantation: Thirty-Sixth Adult Heart Transplantation Report – 2019; focus theme: donor and recipient size match. J Heart Lung Transplant 2019; 38: 1056–1066. doi:10.1016/j.healun.2019.08.004 31548031 PMC6816343

[2] Bos S, Vos R, Van Raemdonck DE, et al. Survival in adult lung transplantation: where are we in 2020? Curr Opin Organ Transplant 2020; 25: 268–273. doi:10.1097/MOT.0000000000000753 32332197

[3] Kwong AJ, Ebel NH, Kim WR, et al. OPTN/SRTR 2021 Annual Data Report: Liver. Am J Transplant 2023; 23: Suppl. 1, S178–S263. doi:10.1016/j.ajt.2023.02.006 37132348

[4] Valapour M, Lehr CJ, Schladt DP, et al. OPTN/SRTR 2021 Annual Data Report: Lung. Am J Transplant 2023; 23: Suppl. 1, S379–S442. doi:10.1016/j.ajt.2023.02.009 37132345 PMC9970343

[5] Colvin M, Smith JM, Ahn Y, et al. OPTN/SRTR 2020 Annual Data Report: Heart. Am J Transplant 2022; 22: Suppl. 2, 350–437. doi:10.1111/ajt.16977 35266620

[6] Ghelichi-Ghojogh M, Ghaem H, Mohammadizadeh F, et al. Graft and patient survival rates in kidney transplantation, and their associated factors: a systematic review and meta-analysis. Iran J Public Health 2021; 50: 1555–1563. doi:10.18502/ijph.v50i8.6801 34917526 PMC8643514

[7] Halverson LP, Hachem RR. Antibody-mediated rejection. Clin Chest Med 2023; 44: 95–103. doi:10.1016/j.ccm.2022.10.008 36774172 PMC10148231

[8] Brugière O, Thabut G, Suberbielle C, et al. Relative impact of human leukocyte antigen mismatching and graft ischemic time after lung transplantation. J Heart Lung Transplant 2008; 27: 628–634. doi:10.1016/j.healun.2008.02.013 18503962

[9] Keogh K, Kaan A, Doran T, et al. HLA mismatching and outcome in heart, heart-lung, and single lung transplantation. J Heart Lung Transplant 1995; 14: 444–451. 7654729

[10] Chalermskulrat W, Neuringer IP, Schmitz JL, et al. Human leukocyte antigen mismatches predispose to the severity of bronchiolitis obliterans syndrome after lung transplantation. Chest 2003; 123: 1825–1831. doi:10.1378/chest.123.6.1825 12796156

[11] Wisser W, Wekerle T, Zlabinger G, et al. Influence of human leukocyte antigen matching on long-term outcome after lung transplantation. J Heart Lung Transplant 1996; 15: 1209–1216. 8981206

[12] Iwaki Y, Yoshida Y, Griffith B. The HLA matching effect in lung transplantation. Transplantation 1993; 56: 1528–1529. 8279029

[13] van den Berg JW, Hepkema BG, Geertsma A, et al. Long-term outcome of lung transplantation is predicted by the number of HLA-DR mismatches. Transplantation 2001; 71: 368–373. doi:10.1097/00007890-200102150-00005 11233895

[14] Hosenpud JD, Edwards EB, Lin HM, et al. Influence of HLA matching on thoracic transplant outcomes. An analysis from the UNOS/ISHLT Thoracic Registry. Circulation 1996; 94: 170–174. doi:10.1161/01.cir.94.2.170 8674175

[15] Quantz MA, Bennett LE, Meyer DM, et al. Does human leukocyte antigen matching influence the outcome of lung transplantation? An analysis of 3,549 lung transplantations. J Heart Lung Transplant 2000; 19: 473–479. doi:10.1016/s1053-2498(00)00081-4 10808155

[16] Peltz M, Edwards LB, Jessen ME, et al. HLA mismatches influence lung transplant recipient survival, bronchiolitis obliterans and rejection: implications for donor lung allocation. J Heart Lung Transplant 2011; 30: 426–434. doi:10.1016/j.healun.2010.10.005 21145259

[17] Hayes D, Whitson BA, Ghadiali SN, et al. Influence of HLA mismatching on survival in lung transplantation. Lung 2015; 193: 789–797. doi:10.1007/s00408-015-9768-9 26220289

[18] Sedgwick P. Multiple hypothesis testing and Bonferroni's correction. BMJ 2014; 349: g6284. doi:10.1136/bmj.g6284 25331533

[19] Firoz A, Kashem M, Zhao H, et al. Human leukocyte antigen mismatch on lung transplantation outcomes. Eur J Cardiothorac Surg 2022; 62: ezac132. doi:10.1093/ejcts/ezac132 35224623

[20] Otten HG, Calis JJA, Keşmir C, et al. Predicted indirectly recognizable HLA epitopes presented by HLA-DR correlate with the *de novo* development of donor-specific HLA IgG antibodies after kidney transplantation. Hum Immunol 2013; 74: 290–296. doi:10.1016/j.humimm.2012.12.004 23232063

[21] Nabulsi S, Otunla AA, Salciccioli J, et al. HLA matching between donors and recipients improves clinical liver transplant graft survival. Liver Int 2024; 44: 411–421. doi:10.1111/liv.15774 38010995

[22] Markus BH, Duquesnoy RJ, Gordon RD, et al. Histocompatibility and liver transplant outcome. Does HLA exert a dualistic effect? Transplantation 1988; 46: 372–377. 3047927 PMC2956422

[23] Wouters EFM. Local and systemic inflammation in chronic obstructive pulmonary disease. Proc Am Thorac Soc 2005; 2: 26–33. doi:10.1513/pats.200408-039MS 16113466

[24] Maher TM. Interstitial lung disease: a review. JAMA 2024; 331: 1655–1665. doi:10.1001/jama.2024.3669 38648021

[25] Courtwright AM, Kamoun M, Kearns J, et al. The impact of HLA-DR mismatch status on retransplant-free survival and bronchiolitis obliterans syndrome-free survival among sensitized lung transplant recipients. J Heart Lung Transplant 2020; 39: 1455–1462. doi:10.1016/j.healun.2020.09.016 33071182

[26] Yoon YM, Velez TE, Upadhyay V, et al. Antigenic responses are hallmarks of fibrotic interstitial lung diseases independent of underlying etiologies. medRxiv 2023; preprint [10.1101/2023.05.08.23289640].

[27] Shaw RJ, Benedict SH, Clark RAF, et al. Pathogenesis of pulmonary fibrosis in interstitial lung disease: alveolar macrophage PDGF(B) gene activation and up-regulation by interferon gamma. Am Rev Respir Dis 1991; 143: 167–173. doi:10.1164/ajrccm/143.1.167 1898843

[28] Holownia A, Wielgat P, Stasiak-Barmuta A, et al. Tregs and HLA-DR expression in sputum cells of COPD patients treated with tiotropium and formoterol. Adv Exp Med Biol 2015; 839: 7–12. doi:10.1007/5584_2014_43 25315616

[29] Faner R, Nuñez B, Sauleda J, et al. HLA distribution in COPD patients. COPD 2013; 10: 138–146. doi:10.3109/15412555.2012.729621 23514216

[30] Scrimini S, Pons J, Cosío B, et al. Expression of HLA-DR in circulating polymorphonuclear neutrophils of COPD patients. Eur Respir J 2014; 42: Suppl. 57, P608. doi:10.1183/13993003/erj.42.Suppl_57.P608

[31] Valapour M, Lehr CJ, Schladt DP, et al. OPTN/SRTR 2023 Annual Data Report: Lung. Am J Transplant 2025; 25: Suppl. 1, S422–S489. doi:10.1016/j.ajt.2025.01.025 39947808 PMC12334194

[32] Lentine KL, Smith JM, Lyden GR, et al. OPTN/SRTR 2023 Annual Data Report: Kidney. Am J Transplant 2025; 25: Suppl. 1, S22–S137. doi:10.1016/j.ajt.2025.01.020 39947805 PMC12414513

[33] Sureshkumar KK, Chopra B. Induction type and outcomes in HLA-DR mismatch kidney transplantation. Transplant Proc 2019; 51: 1796–1800. doi:10.1016/j.transproceed.2019.04.059 31399165

[34] Kleid L, Walter J, Moehnle P, et al. High-risk HLA-DQ mismatches are associated with adverse outcomes after lung transplantation. Transpl Int 2024; 37: 13010. doi:10.3389/ti.2024.13010 39381015 PMC11460317

[35] Locke AF, Hickey M, Valenzuela NM, et al. Virtual and reality: an analysis of the UCLA virtual crossmatch exchanges. Transplantation 2023; 107: 1776. doi:10.1097/TP.0000000000004586 36944607 PMC10358445

