# Supplementary material for: The influence of HLA matching on graft survival in lung transplant recipients is indication specific: a UNOS database analysis
Source: Eur Respir J. 2026 Sep 8;68(3):2501890. doi: 10.1183/13993003.01890-2025 (PMC13550823; doi:10.1183/13993003.01890-2025)
Supplement: Supplementary file 1 [file ERJ-01890-2025.Supplement.pdf]

Supplementary Materials

Supplementary table 1. Multivariate cox survival analysis stratified by indication, complete case analysis n = 27,140.

Variable	1 years		5 years		10 years	
	HR (95%, CI)	P-Value	HR (95%, CI)	P-Value	HR (95%, CI)	P-Value
**COPD**						
Overall mismatch (0-2 vs 3-6 mismatches)	5.81 (1.44-23.38)	0.013	1.63 (1.14-2.35)	0.008	1.50 (1.12-2.01)	0.006
HLA-A (2 mismatches vs 0 reference)	0.86 (0.55-1.34)	0.508	1.06 (0.83-1.35)	0.656	1.12 (0.92-1.38)	0.258
HLA-B (2 mismatches vs 0 reference)	2.65 (0.65-10.72)	0.171	1.41 (0.89-2.24)	0.141	1.21 (0.84-1.74)	0.309
HLA-DR (2 mismatches vs 0 reference)	3.57 (1.32-9.66)	0.012	1.19 (0.89-1.59)	0.251	1.06 (0.83-1.37)	0.624
**Interstitial Pneumonia**						
Overall mismatch (0-2 vs 3-6 mismatches)	1.25 (0.30-5.23)	0.763	1.58 (0.57-4.35)	0.375	1.06 (0.45-2.51)	0.892
HLA-A (2 mismatches vs 0 reference)	0.90 (0.35-2.36)	0.835	0.94 (0.52-1.67)	0.823	1.05 (0.60-1.85)	0.862
HLA-B (2 mismatches vs 0 reference)	n/a	n/a	2.26 (0.31-16.55)	0.421	1.46 (0.34-6.38)	0.611
HLA-DR (2 mismatches vs 0 reference)	1.38 (0.48-4.00)	0.549	1.14 (0.63-2.07)	0.674	1.40 (0.75-2.64)	0.292
**Bronchiectasis**						
Overall mismatch (0-2 vs 3-6 mismatches)	0.87 (0.58-1.30)	0.498	1.24 (0.94-1.63)	0.124	1.14 (0.89-1.45)	0.298
HLA-A (2 mismatches vs 0 reference)	1.04 (0.75-1.43)	0.821	1.24 (1.02-1.51)	0.035	1.05 (0.88-1.27)	0.576
HLA-B (2 mismatches vs 0 reference)	0.65 (0.41-1.04)	0.072	0.88 (0.63-1.24)	0.469	0.77 (0.57-1.05)	0.094
HLA-DR (2 mismatches vs 0 reference)	0.96 (0.66-1.41)	0.848	1.28 (1.01-1.61)	0.039	1.14 (0.92-1.42)	0.231
**Pulmonary Arterial Hypertension**						

Overall mismatch (0-2 vs 3-6 mismatches)	0.98 (0.40-2.42)	0.962	0.83 (0.57-1.21)	0.3335 5023	1.00 (0.71-1.39)	0.9802 9131
HLA-A (2 mismatches vs 0 reference)	0.97 (0.48-1.96)	0.923	0.96 (0.71-1.29)	0.786	1.11 (0.85-1.46)	0.431
HLA-B (2 mismatches vs 0 reference)	1.16 (0.28-4.80)	0.842	0.93 (0.52-1.67)	0.806	1.02 (0.63-1.65)	0.933
HLA-DR (2 mismatches vs 0 reference)	1.30 (0.56-3.02)	0.539	0.81 (0.58-1.12)	0.207	0.90 (0.67-1.21)	0.493

Tripod Checklist.

TRIPOD Checklist: Prediction Model Development

Section/Topic	Item	Checklist Item	Page
**Title and abstract**			
Title	1	Identify the study as developing and/or validating a multivariable prediction model, the target population, and the outcome to be predicted.	
Abstract	2	Provide a summary of objectives, study design, setting, participants, sample size, predictors, outcome, statistical analysis, results, and conclusions.	
**Introduction**			
Background and objectives	3a	Explain the medical context (including whether diagnostic or prognostic) and rationale for developing or validating the multivariable prediction model, including references to existing models.	
	3b	Specify the objectives, including whether the study describes the development or validation of the model or both.	
**Methods**			
Source of data	4a	Describe the study design or source of data (e.g., randomized trial, cohort, or registry data), separately for the development and validation data sets, if applicable.	
	4b	Specify the key study dates, including start of accrual; end of accrual; and, if applicable, end of follow-up.	
Participants	5a	Specify key elements of the study setting (e.g., primary care, secondary care, general population) including number and location of centres.	
	5b	Describe eligibility criteria for participants.	
	5c	Give details of treatments received, if relevant.	
Outcome	6a	Clearly define the outcome that is predicted by the prediction model, including how and when assessed.	
	6b	Report any actions to blind assessment of the outcome to be predicted.	
Predictors	7a	Clearly define all predictors used in developing or validating the multivariable prediction model, including how and when they were measured.	
	7b	Report any actions to blind assessment of predictors for the outcome and other predictors.	
Sample size	8	Explain how the study size was arrived at.	
Missing data	9	Describe how missing data were handled (e.g., complete-case analysis, single imputation, multiple imputation) with details of any imputation method.	
Statistical analysis methods	10a	Describe how predictors were handled in the analyses.	
	10b	Specify type of model, all model-building procedures (including any predictor selection), and method for internal validation.	
	10d	Specify all measures used to assess model performance and, if relevant, to compare multiple models.	
Risk groups	11	Provide details on how risk groups were created, if done.	
**Results**			
Participants	13a	Describe the flow of participants through the study, including the number of participants with and without the outcome and, if applicable, a summary of the follow-up time. A diagram may be helpful.	
	13b	Describe the characteristics of the participants (basic demographics, clinical features, available predictors), including the number of participants with missing data for predictors and outcome.	
Model development	14a	Specify the number of participants and outcome events in each analysis.	
	14b	If done, report the unadjusted association between each candidate predictor and outcome.	
Model specification	15a	Present the full prediction model to allow predictions for individuals (i.e., all regression coefficients, and model intercept or baseline survival at a given time point).	
	15b	Explain how to the use the prediction model.	
Model performance	16	Report performance measures (with CIs) for the prediction model.	
**Discussion**			
Limitations	18	Discuss any limitations of the study (such as nonrepresentative sample, few events per predictor, missing data).	
Interpretation	19b	Give an overall interpretation of the results, considering objectives, limitations, and results from similar studies, and other relevant evidence.	
Implications	20	Discuss the potential clinical use of the model and implications for future research.	
**Other information**			
Supplementary information	21	Provide information about the availability of supplementary resources, such as study protocol, Web calculator, and data sets.	
Funding	22	Give the source of funding and the role of the funders for the present study.	

We recommend using the TRIPOD Checklist in conjunction with the TRIPOD Explanation and Elaboration document.

Strobe checklist.

STROBE Statement—checklist of items that should be included in reports of observational studies

	Item No.	Recommendation
**Title and abstract**	1	(*a*) Indicate the study's design with a commonly used term in the title or the abstract
		(*b*) Provide in the abstract an informative and balanced summary of what was done and what was found
**Introduction**		
Background/rationale	2	Explain the scientific background and rationale for the investigation being reported
Objectives	3	State specific objectives, including any prespecified hypotheses
**Methods**		
Study design	4	Present key elements of study design early in the paper
Setting	5	Describe the setting, locations, and relevant dates, including periods of recruitment, exposure, follow-up, and data collection
Participants	6	(*a*) *Cohort study*—Give the eligibility criteria, and the sources and methods of selection of participants. Describe methods of follow-up<br>*Case-control study*—Give the eligibility criteria, and the sources and methods of case ascertainment and control selection. Give the rationale for the choice of cases and controls<br>*Cross-sectional study*—Give the eligibility criteria, and the sources and methods of selection of participants
		(*b*) *Cohort study*—For matched studies, give matching criteria and number of exposed and unexposed<br>*Case-control study*—For matched studies, give matching criteria and the number of controls per case
Variables	7	Clearly define all outcomes, exposures, predictors, potential confounders, and effect modifiers. Give diagnostic criteria, if applicable
Data sources/ measurement	8*	For each variable of interest, give sources of data and details of methods of assessment (measurement). Describe comparability of assessment methods if there is more than one group
Bias	9	Describe any efforts to address potential sources of bias
Study size	10	Explain how the study size was arrived at

Continued on next page

Quantitative variables	11	Explain how quantitative variables were handled in the analyses. If applicable, describe which groupings were chosen and why
Statistical methods	12	(*a*) Describe all statistical methods, including those used to control for confounding
		(*b*) Describe any methods used to examine subgroups and interactions
		(*c*) Explain how missing data were addressed
		(*d*) *Cohort study*—If applicable, explain how loss to follow-up was addressed<br>*Case-control study*—If applicable, explain how matching of cases and controls was addressed<br>*Cross-sectional study*—If applicable, describe analytical methods taking account of sampling strategy
		(*e*) Describe any sensitivity analyses
**Results**		
Participants	13*	(a) Report numbers of individuals at each stage of study—eg numbers potentially eligible, examined for eligibility, confirmed eligible, included in the study, completing follow-up, and analysed
		(b) Give reasons for non-participation at each stage
		(c) Consider use of a flow diagram
Descriptive data	14*	(a) Give characteristics of study participants (eg demographic, clinical, social) and information on exposures and potential confounders
		(b) Indicate number of participants with missing data for each variable of interest
		(c) *Cohort study*—Summarise follow-up time (eg, average and total amount)
Outcome data	15*	*Cohort study*—Report numbers of outcome events or summary measures over time
		Case-control study—Report numbers in each exposure category, or summary measures of exposure
		Cross-sectional study—Report numbers of outcome events or summary measures
Main results	16	(*a*) Give unadjusted estimates and, if applicable, confounder-adjusted estimates and their precision (eg, 95% confidence interval). Make clear which confounders were adjusted for and why they were included
		(*b*) Report category boundaries when continuous variables were categorized
		(*c*) If relevant, consider translating estimates of relative risk into absolute risk for a meaningful time period

Continued on next page

Other analyses	17	Report other analyses done—eg analyses of subgroups and interactions, and sensitivity analyses
**Discussion**		
Key results	18	Summarise key results with reference to study objectives
Limitations	19	Discuss limitations of the study, taking into account sources of potential bias or imprecision. Discuss both direction and magnitude of any potential bias
Interpretation	20	Give a cautious overall interpretation of results considering objectives, limitations, multiplicity of analyses, results from similar studies, and other relevant evidence
Generalisability	21	Discuss the generalisability (external validity) of the study results
**Other information**		
Funding	22	Give the source of funding and the role of the funders for the present study and, if applicable, for the original study on which the present article is based

*Give information separately for cases and controls in case-control studies and, if applicable, for exposed and unexposed groups in cohort and cross-sectional studies.

Note: An Explanation and Elaboration article discusses each checklist item and gives methodological background and published examples of transparent reporting. The STROBE checklist is best used in conjunction with this article (freely available on the Web sites of PLoS Medicine at http://www.plosmedicine.org/, Annals of Internal Medicine at http://www.annals.org/, and Epidemiology at http://www.epidem.com/). Information on the STROBE Initiative is available at www.strobe-statement.org.
